# Involvement of the TGF-β Signaling Pathway in the Development of YAP-Driven Osteosarcoma Lung Metastasis

**DOI:** 10.3389/fonc.2021.765711

**Published:** 2021-10-26

**Authors:** Sarah Morice, Geoffroy Danieau, Robel Tesfaye, Mathilde Mullard, Régis Brion, Maryne Dupuy, Benjamin Ory, Bénédicte Brounais-Le Royer, Isabelle Corre, Françoise Redini, Franck Verrecchia

**Affiliations:** ^1^ Université de Nantes, Institut National de la Santé et de la Recherche Médicale Unité Mixte de Recherche (INSERM UMR) 1238, Nantes, France; ^2^ Centre Hospitalier Universitaire (CHU) Hôtel Dieu, Nantes, France

**Keywords:** osteosarcoma, lung metastases, Hippo, YAP, TEAD, TGF-β/Smad3

## Abstract

**Background:**

The poor survival rate of patients with osteosarcoma (OS), specifically with metastases at diagnosis, undergoes the urgency to develop new therapeutic strategies. Although we recently demonstrated the key role of YAP/TEAD signaling in the growth of OS primary tumor, the molecular mechanisms by which YAP regulates metastases development remain poorly understood.

**Methods:**

The molecular mechanisms by which YAP regulates metastases development were studied using an overexpression of mutated forms of YAP able or not able to interact with TEAD. Molecular signatures were identified using RNA-sequencing analysis and gene set enrichment. Interactions between YAP and Smad3 were studied using proximity ligation assay (PLA), immunoprecipitation, and promoter/specific gene assays. The involvement of the TGF-β pathway in the ability of YAP to stimulate metastatic development *in vivo* was studied using an inhibitor of the TGF-β cascade in a preclinical model of OS and *in vitro* on the ability of OS cells to migrate and invade.

**Results:**

Our work shows that a high YAP expression is associated with the presence of lung metastases which predicts a poor prognosis. Molecular analysis indicates that TGF-β signaling is involved in YAP-driven osteosarcoma cell pro-migratory phenotype, epithelial mesenchymal transition, cell migration, and *in vivo* lung metastasis development. Regardless of its ability to bind to TEAD, YAP interacts with Smad3 and stimulates the transcriptional activity of TGF-β/Smad3, thereby enhancing the ability of TGF-β to stimulate lung metastasis development.

**Conclusions:**

We demonstrated the crucial involvement of the TGF-β/Smad3 signaling pathway in YAP-driven lung metastasis development in OS.

## 1 Introduction

Osteosarcoma (OS) is the most prevalent primary malignant bone tumor and the third most frequent cancer in children and adolescents (1). The diagnosis of OS is supported by clinical and radiological examinations and a histological evaluation of biopsies (2). OS usually originates in the metaphysis of the long bones and is marked by the production of osteoid matrix by mesenchymal cells. Although some predisposing genetic factors, such as mutations in p*53* gene, have been found, the exact etiology of this disease remains unclear (1).

Despite the introduction of neoadjuvant and adjuvant chemotherapy following surgical tumor resection, metastatic relapse rates have remained unchanged over the last four decades (3). In addition, the presence of metastases at diagnosis is the leading cause of patient deaths, with a 5-year survival rate of approximately 20 *versus* 70–75% for patients with localized disease (1, 2, 4). Unfortunately, while only 15 to 20% of patients have clinically detectable pulmonary metastases at diagnosis, it is suspected that 80 to 90% of them have undetectable micrometastases (5). Therefore, a better understanding of OS biology is required to treat metastatic OS.

The Hippo cascade, originally identified in *Drosophila* (6), is activated by a variety of signals and plays a critical role in the regulation of tumor cellular processes, such as cell proliferation, apoptosis, and migration (6, 7). Schematically, following the activation of the Hippo signaling pathway, activated MST1/2 (mammalian Ste20-like kinases 1/2) phosphorylates and activates LATS1/2 factors (large tumor suppressor 1/2), resulting in the inhibition of YAP/TAZ (Yes-Associated Protein/paralog transcriptional coactivator with PDZ-binding motif). The phosphorylation of YAP on Ser127 allows its binding with the 14-3-3 protein, leading to its cytoplasmic sequestration and to its degradation *via* the ubiquitin–proteasome pathway (8). When the Hippo signaling pathway is not engaged, neither YAP nor TAZ is phosphorylated, and thus they can be translocated into the nucleus where they act as co-transcriptional factors. The major DNA-binding partners of YAP are TEAD1–4 (TEA-domain DNA-binding transcription factor 1–4) proteins capable of regulating target genes that control key cellular process, including proliferation, differentiation, and apoptosis (9).

Increasing evidence has shown that the disruption of the Hippo cascade or the anomalous activation of YAP/TAZ is tightly associated with cancers such as lung, prostate, breast, liver, stomach, pancreatic, and brain cancers (7, 10–13). In this context, YAP/TAZ have been shown to be implicated in different stages of cancer development, such as cancer initiation, progression, and metastasis. For OS, tissue array and RNAseq analysis demonstrated a YAP signature in OS patients and a high level of YAP protein expression in tumor tissue (14, 15). Interestingly, this overexpression of YAP is linked to poor prognosis (14, 16). The molecular mechanisms behind YAP overexpression in OS appear to be complex, but evidence indicates that it may be due, in a large part, to the stem cell transcription factor SOX2 (17). Recently, using mutated forms of YAP able to interact with TEAD or not, we demonstrated the critical involvement of TEAD in YAP-driven OS primary tumor growth (14). However, the role of YAP in OS lung metastatic development and the molecular mechanisms by which YAP would be able to regulate the OS lung metastatic process have yet to be elucidated. Using *in vitro* and *in vivo* techniques, the aims of this work were to (1) determine the role of YAP in OS lung metastatic process, (2) identify the transcriptional partners of YAP implicated in YAP-driven lung metastatic development, and (3) test the effect of the YAP inhibitor, verteporfin, in OS lung metastatic development.

## 2 Materials and Methods

### 2.1 Osteosarcoma Mouse Model

Four-week-old female Rj : NMRI-nude mice (Janvier, Le Genest Saint Isle, France) were housed under pathogen-free conditions at the Experimental Therapy Unit of Nantes University (France) in accordance with the institutional guidelines of the French Ethical Committee (CEEA Pays de la Loire no. 06: project authorization 8405). The mice were anesthetized by inhalation of isoflurane before receiving an intramuscular injection of 1.10^6^ K-HOS parental or mutant cells near the tibia. For the *in vivo* experiments using SD-208 or verteporfin, at 5 days after the cell injections, groups of mice received 60 or 20 mg/kg SD-208 or the control vehicle twice a week by intraperitoneal injection. The mice were sacrificed when the tumor volume reached 1,000 mm^3^, and lung metastases were analyzed and counted in each group of mice.

### 2.2 Cell Culture and Reagents

As previously described (14), OS (K-HOS, HOS, MG63, and G292) cell lines and HEK cells were purchased from ATCC (LGC Standards, Molsheim, France) and from Invitrogen (Thermo Fisher, Courtaboeuf, France), respectively. The cells were cultured in high-glucose Dulbecco’s modified Eagle’s medium (DMEM, Lonza, Basel, Switzerland) supplemented with 10% fetal calf serum (FCS, Hyclone Perbio, Bezons, France). The authentication of cell lines used in the research has been realized by PCR single-locus technology (Eurofins, Ebersberg, Germany). The mycoplasma level has been tested according to the protocol of the manufacturer (Lonza, Basel, Switzerland).

As previously described (14), mutant YAP-S94A- and YAP-S127A-expressing cells were generated using retrovirus infection by transfecting 293 Phoenix retrovirus packaging cells with an empty vector, pQCXIH-Myc-YAP-S94A and pQCXIH-Flag-YAP-S127A (gifts from Kunliang Guan, plasmid #33094 and #33092; http://n2t.net/addgene:33094; RRID : Addgene-33094 and -33092).

SD208 and verteporfin were purchased from Sigma (Sigma-Aldrich, St. Quentin-Fallavier, France) and Tocris (Bristol, UK) and dissolved in dimethyl sulfoxide (DMSO, Sigma-Aldrich, St. Quentin-Fallavier, France). For the *in vivo* experiments, SD 208 or verteporfin was first dissolved in DMSO and further diluted in saline medium at a maximal dose of 2% DMSO v/v. TGF-β1 was purchased from R&D Systems, Inc. (Minneapolis, MN).

### 2.3 Luciferase Reporter Assay and Plasmid Constructs

Transient cell transfections were performed with jetPEI™ (Polyplus transfection, Illkirch, France). The pRLTK-Renilla luciferase expression vector was co-transfected in all experiments to monitor transfection efficiencies. Luciferase activity was determined with the dual-Luciferase reporter assay system (Promega, Charbonnieres, France). The (CAGA)_9_-Lux was used as reporter constructs specific for Smad3/4-driven signaling (18). The 8xGTIIC-Luc (gift from Stefano Piccolo, Addgene plasmid #34615; http://n2t.net/addgene:34615; RRID : Addgene-34615; (TEAD)_8_-lux in the text) construct was used as a reporter construct specific for TEAD-driven signaling.

### 2.4 Real-Time Migration Assay

OS cell migration was assessed using xCELLigence RTCA DP (ACEA Biosciences) using an electronically integrated Boyden chamber (CIM-Plate^®^ 16). A total of 30 × 10^3^ OS cells were seeded in CIM-Plate^®^ 16; migration was measured over the course of 24 h, and the experiment was run in triplicate and repeated three times.

### 2.5 Histologic Analysis and Immunohistochemistry

The lungs were fixed in 10% buffered formaldehyde and embedded in paraffin. The lung sections (3 μm in thickness) were mounted on glass slides and stained with hematoxylin–eosin. OS bone tumors were fixed in 10% buffered formaldehyde and embedded in paraffin.

### 2.6 RNA Extraction and Real-Time Polymerase Chain Reaction

Total RNA was extracted from cells and tumors using NucleoSpin^®^RNAplus (Macherey Nagel, Duren, Germany) and reverse-transcribed using the Maxima H minus first-strand cDNA synthesis kit (Thermo Fisher, Courtaboeuf, France). Real-time PCR was performed using DNA primers (the primer sequences are available in **Table 1**) using QuantStudio 7 Flex Real-Time PCR System (Thermo Fisher, Courtaboeuf, France) with SYBR^®^ Select Master Mix (Life Technologies, Carlsbad, CA). The target gene expression was normalized to glyceraldehyde 3-phosphatedehydrogenase and β-actin.

**Table 1:** Primer sequences for quantitative RT-PCR.

Gene	Forward	Reverse
**c-myc**	CACCAGCAGCGACTCTGA	GATCCAGACTCTGACCTTTTGC
**Fosl1**	GGCCTCTGACCTACCCTCA	CTTCCTCCGGGCTGATCT
**VEGFa**	CCTTGCTGCTCTACCTCCAC	CCACTTCGTGATGATTCTGC
**CBFA1**	GCCTAGGCGCATTTCAGA	GCTCTTCTTACTGAGAGTGGAAGG
**AKT1**	TACGAGAAGAAGCTCAGCCC	TTGGTCAGGTGGTGTGATGG
**Gli1**	CCAACTCCACAGGCATACAGGAT	CACAGATTCAGGCTCACGCTTC
**GAPDH**	TGGGTGTGAACCATGAGAAGTATG	GGTGCAGGAGGCATTGCT
**OPN**	GCCGAGGTGATAGTGTGGTT	TGAGGTGATGTCCTCGTCTG
**OPG**	CAGCTCACAAGAACAGACTTTCC	TCGAAGGTGAGGTTAGCATGTC
**RANK**	TTCTGCTTCTCTTCGCGTCT	CCAGTGCCACAAATTAGCTGT
**YAP1**	TGACCCTCGTTTTGCCATGA	GTTGCTGCTGGTTGGAGTTG
**Cyr61**	CCAGTGTACAGCAGCCTGAA	GGCCGGTATTTCTTCACACTC
**CTGF**	AGGAGTGGGTGTGTGACGAG	CGGGACAGTTGTAATGGCAG
**PAI1**	AGATCGAGGTGAACGAGAGTGGCACG	TTTGTCCCAGATGAAGGCGTCTTTCC
**SNAIL**	AATCCAGAGTTTACCTTCCAGCA	TCCCAGATGAGCATTGGCAG
**TWIST2**	CTCAGCTACGCCTTCTCCG	CGACGGACAGCCCTGG
**Vimentin**	TGGTCTAACGGTTTCCCCTA	GACCTCGGAGCGAGAGTG
**CDH2**	AGGCTTCTGGTGAAATCGCA	GCAGTTGCTAAACTTCACATTGAG
**MMP2**	AGAAGGCTGTGTTCTTTGCAG	AGGCTGGTCAGTGGCTTG
**MMP9**	GACCAAGGGATGGGGGATC	CTTGACAGGCAAGTGCTGAC
**MMP1**	CCTACGTACCCACACACAGC	ACATTGGCCTTGATCTCAGC
**PDGFb**	GAGTTGGACCTGAACATGAC	GTTGGTGCGGTCTATGAG
**CXCR4**	CCGAGGAAATGGGCTCAGGGG A	TGATGGAGTAGATGGTGGGCAGGA
**Col1a1**	CTGGACCTAAAGGTGCTGCT	GCTCCAGCCTCTCCATCTTT

### 2.7 Gelatin Zymography Assay

Cells were cultured without serum for 48 h, and conditioned media were analyzed by gelatin zymography in 10% polyacrylamide gels containing 1 mg/ml gelatin (Sigma-Aldrich, St. Quentin-Fallavier, France).

### 2.8 Western Blot Analysis

Cells were lysed in lysis buffer (SDS 1%; Tris, pH 7.4, 10 mM; and sodium orthovanadate 1 mM), and protein concentration was determined using the BCA Protein Assay Kit (Sigma-Aldrich, St. Quentin-Fallavier, France). Samples in Laemmli buffer (62.5 mM Tris–HCl, pH 6.8, 2% SDS, 10% glycerol, 5% 2-mercaptoethanol, and 0.001% bromophenol blue) containing equal amounts of total protein extracts were separated by SDS-polyacrylamide gel electrophoresis (SDS-PAGE) and transferred to polyvinylidene fluoride transfer membrane (Thermo Scientific, Illkirch, France). The antibodies used for western blotting were YAP1 (1:8,000; Proteintech, 1358-AP), anti-Flag (1:1,000, Sigma Aldrich, F1804), myc-tag (1:1,000, cell signaling, 2276), HA-tag (1:500; Cell Signaling, 2367), β-actin (1:1,000; Cell Signaling, 8457), anti-mouse IgG-HRP (1:10,000, Santa Cruz Biotechnology, sc-2314), and anti-rabbit IgG-HRP (1:10,000; Santa Cruz Biotechnology, sc-2004). Antibody binding was visualized with the enhanced chemiluminescence system (SuperSignal West Pico Chemiluminescent Substrate, Thermo Scientific, Illkirch, France). For quantification, luminescence was detected with a Charge Couple Device camera and analyzed using the GeneTools program (Syngene, Cambridge, United Kingdom).

### 2.9 Immunoprecipitation

HEK239 cells were transfected with vectors pCMV-Flag-YAP-S94A and pCMV-Flag-S127A-YAP (gifts from Kunliang Guan, Addgene plasmids #33055, #33102 and #27370; RRID: Addgene-33055, -33102 and -27370) and Smad3-Myc expression vector (19).

At 24 h after transfection, media were changed with fresh DMEM containing 1% FCS for 24 h in the presence of TGF-β1 (5 ng/ml) for 45 min. Cells were then lysed in IP-lysis buffer (Invitrogen, Courtaboeuf, France). Equal amounts of proteins were precleared overnight at 4°C using Protein-A/G-agarose (Santa Cruz Biotechnology, CA) and incubated with primary antibody against Flag (4 µg/ml; Sigma Aldrich, F7425) and HA-tag (1:50; Cell Signaling, 3724S) for 2 h at 4°C. Moreover, 50 µl of Protein-A/G-agarose was then added and incubated overnight at 4°C. The beads were washed three times with IP-lysis buffer; thereafter, 30 µl of Laemmli buffer was added and boiled for 5 min. After centrifugation, supernatants were harvested and processed for SDS-PAGE and Western blot as described above.

### 2.10 *In Situ* Proximity Ligation Assay, Immunofluorescence, and Confocal Microscopy

#### 2.10.1 Duolink PLA^®^

A total of 5 × 10^3^ OS cells were seeded in Ibidi µ-Slide VI 0.4 during 25 h. The medium was then replaced with DMEM. At 24 h after, TGF-β1 (5 ng/ml) was either added for 45 min or not. The cells were then fixed with 4% PFA for 15 min at room temperature and incubated overnight at 4°C with primary antibody against YAP (1:100; Cell Signaling, 14074) and/or Smad3 (1:1,000, Abcam, ab40854). *In situ* PLA was performed using DuoLink in Situ Reagents (Sigma-Aldrich, St. Quentin-Fallavier, France) according to the protocol of the manufacturer.

#### 2.10.2 Immunofluorescence Assays

Cells were seeded onto Ibidi µ-Slide 8 Well overnight, fixed with 4% paraformaldehyde for 15 min, and permeabilized with 0.5% Triton. The samples were incubated with anti-vinculin−FITC antibody (Sigma-Aldrich, St. Quentin-Fallavier, France) for 2 h at room temperature (1:200) and then washed in phosphate-buffered saline. F-actin and the nucleus were stained using Alexa-fluor 488 phalloidin (1:1,000) and DAPI (1:1,000), respectively. The images were acquired using a confocal microscope (NIKON A1 N-SIM) and processed using ImageJ.

### 2.11 RNA-seq and Analysis

Library preparation and sequencing were performed at Active Motif, Inc. Libraries were prepared from purified RNA using the Illumina TruSeq Stranded mRNA Sample Preparation kit, and sequencing was done on Illumina NextSeq 500 as 42-nt-long paired-end reads. Read mapping and fragment quantification for each gene were also performed at Active Motif. Briefly, read mapping against human genome (GRCh38) was done using STAR algorithm (20) with default settings, and fragment assignment was done using feature counts with gene annotations from Subread package. Only read pairs having both ends aligned with a minimum overlap of 25 base pairs and mapping to the same chromosome and on the same strand were counted (featureCounts -p -B -C -minOverlap 25). Differential gene expression analysis was performed using DESeq2 package. The *p*-values obtained were corrected for false positives by using independent hypothesis weighting (package IHW) multiple testing adjustment method. Genes were considered significantly differentially expressed if log2 fold change was over 1 or less than -1, and the false discovery rate was less than 0.05. For the differentially expressed genes, over-representation and gene set enrichment analysis (GSEA) were done using clusterProfiler package, and the results were plotted using enrich Plot.

Further GSEA was performed using GSEA software (http://software.broadinstitute.org/gsea/). The gene sets used are described in **Table 2**.

**Table 2:** Gene set enrichment analysis.

Gene set	Link
**Focal adhesion**	http://software.broadinstitute.org/gsea/msigdb/geneset_page.jsp?geneSetName=KEGG_FOCAL_ADHESION&keywords=cell%20migration
**GO positive regulation of cell migration GO:0030335**	http://www.informatics.jax.org/vocab/gene_ontology/GO:0030335
**Early response to TGF-β**	http://software.broadinstitute.org/gsea/msigdb/geneset_page.jsp?geneSetName=VERRECCHIA_EARLY_RESPONSE_TO_TGFB1&keywords=verrecchia
**Delayed response to TGF-β**	http://software.broadinstitute.org/gsea/msigdb/geneset_page.jsp?geneSetName=VERRECCHIA_DELAYED_RESPONSE_TO_TGFB1&keywords=verrecchia
**GO response to transforming growth factor beta GO:0071559**	http://www.informatics.jax.org/vocab/gene_ontology/GO:0071559
**Reactome extracellular matrix organization R-HSA-1474244**	http://www.reactome.org/content/detail/R-HSA-1474244

### 2.12 Statistical Analysis

Histogram and data are shown as mean ± SD of a minimum of three independent experiments.

Statistical analyses were performed using GraphPad Prism, version, 6 for Windows (GraphPad Software, La Jolla, CA), (www.graphpad.com). The Wilcoxon matched test was used to compare the expression levels between OS and matched normal tissue. The Mann–Whitney test was used to compare the difference between the two groups. A *p*-value under or equal to 0.05 was considered statistically significant.

### 2.13 Database

Kaplan–Meier analysis of osteosarcoma patient tumor samples was performed using the R2 Genomics Analysis and Visualization Platform. The genome-wide gene expression analyses of high-grade osteosarcoma are from GSE42352 (https://www.ncbi.nlm.nih.gov/geo/query/acc.cgi?acc=GSE42352).

The datasets supporting the current study have been deposited in the public repository GSE184003 (https://www.ncbi.nlm.nih.gov/geo/query/acc.cgi?acc=GSE184003).

## 3 Results

### 3.1 YAP Promotes *In Vivo* Lung Metastatic Progression in OS

**Figure 1:**
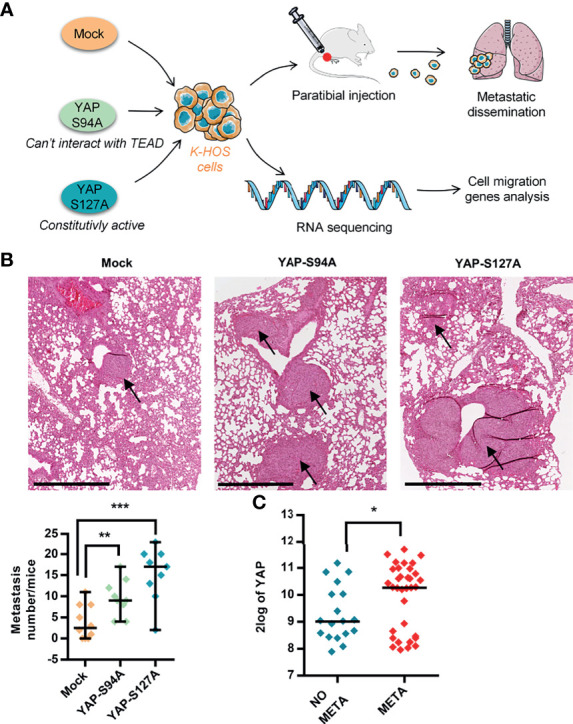
Role of YAP in osteosarcoma (OS) lung metastasis development. **(A)** Schematic illustration of the design of the experimental protocols. K-HOS cells were transduced with mock-, YAPS94A-, or YAPS127A-vector and stably selected. Intramuscular paratibial injections of these cells were performed in nude mice, and the lung metastasis development was followed. Parallel to this, RNAseq analysis was carried out on the YAP mutated cells. **(B)** Intramuscular paratibial injection of 1.10^6^ mock-, YAPS94A-, and YAPS127A-transfected K-HOS cells was performed in three groups of 12 nude mice. The number of lung metastases was counted 28 days after injection of the cells (left panel, mean ± SD; **p < 0.01, ***p < 0.001). Photographs showing typical hematoxylin and eosin staining of the lungs for each group (right panel). Bar = 500 µm. **(C)** Relative YAP gene expression in OS patients with (META) or without (NO META) metastases at diagnosis following bioinformatics analysis of RNAseq data seq GSE42352 (21) from an OS patient cohort comprising 53 samples. Analysis was performed using R2 (http://r2.amc.nl). *p < 0.05.

Recently, using mutated forms of YAP capable to bind TEAD (YAPS127A) or not (YAPS94A), we demonstrated the key role of TEAD in YAP-induced primary OS tumor growth (14). To fully comprehend the role of YAP in OS progression, we have now examined the effect of YAP overexpression on pulmonary metastasis progression, as depicted in **Figure 1A**. Of note is that the expression and functionality of the mutated forms of YAP in OS cell lines were previously demonstrated (14). As shown in **Figure 1B**, the overexpression of both mutated forms of YAP significantly increased the number of pulmonary metastases in a mice preclinical model of OS. Although the number of lung metastases is greater in response to the expression of a YAP protein able to interact with TEAD (**Figure 1B**; YAPS127A- *vs.* mock-transfected samples), we observe that the expression of a YAP unable to interact with TEAD also stimulates the development of lung metastases (**Figure 1B**; YAPS94A- *vs.* mock-transfected samples), suggesting that two mechanisms, one TEAD dependent and one TEAD independent, are involved in the YAP-driven OS metastatic process.

To validate the medical relevance of these observations, we next analyzed RNAseq data from the biopsies of patients. As shown in **Figure 1C**, the data demonstrate that YAP is overexpressed in OS patients with metastases compared with OS patients without metastases.

Taken together, these findings demonstrate (a) that YAP stimulates OS lung metastasis development, (b) that two mechanisms, one TEAD dependent and one TEAD independent, may be involved in the YAP-driven OS metastatic process, and (c) a correlation between high YAP expression and the presence of metastases in OS patients.

### 3.2 YAP Promotes Pro-Migratory Cell Phenotype

**Figure 2:**
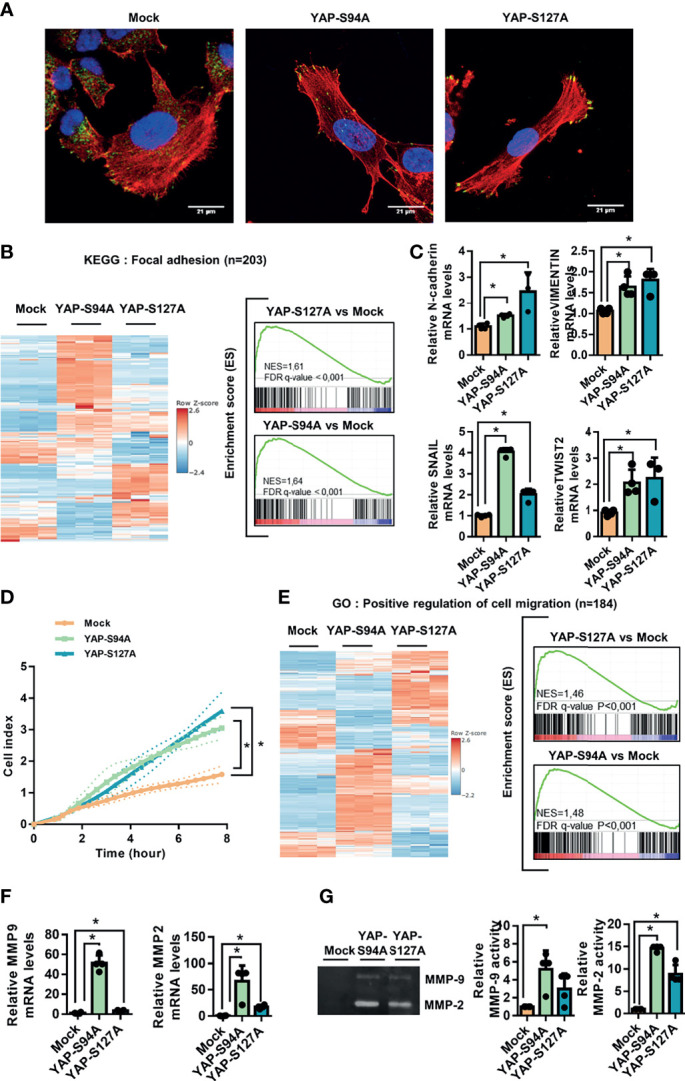
Involvement of YAP in osteosarcoma cell migration. **(A)** Cultures of mock-, YAPS94A-, and YAPS127A-transfected cells were fixed, permeabilized, and stained with a monoclonal antibody raised against vinculin (green). F-actin cytoskeleton and nuclei were respectively stained by phalloidine (red) and DAPI labeling (blue). Representative photographs of three independent experiments are shown. **(B)** Heat map showing the mRNA levels of 203 genes involved in focal adhesion (left panel). Enrichment score (ES) plots of gene set enrichment analysis (GSEA) show a significant upregulation of genes involved in focal adhesion in YAPS94A- and YAPS127A-transfected K-HOS cells compared to mock-transfected cells (right panel). The color scales are based on Z-scores. **(C)** N-cadherin, SNAIL, TWIST2, and vimentin mRNA steady-state levels were quantified by RT-qPCR analysis in mock-, YAPS94A-, and YAPS127A-transfected K-HOS cells. Bars indicate the means ± SD of three independent experiments, each performed in duplicate (*p < 0.05). **(D)** Real-time migration assays using Xcelligence technology were done to compare the cell migration rate between mock-, YAPS94A-, and YAPS127A-transfected K-HOS cells. Each point indicates the means ± SD of three independent experiments, each performed in sextuplicate (*p < 0.05). **(E)** Heat map showing the mRNA levels of 184 genes involved in cell migration (left panel). The ES plots of GSEA show a significant upregulation of genes involved in cell migration in YAPS94A- and YAPS127A-transfected K-HOS cells compared to mock-transfected cells (right panel). The color scales are based on Z-scores. **(F)** MMP-2 and MMP-9 mRNA steady-state levels were quantified by RT-q-PCR analysis in mock-, YAPS94A-, and YAPS127A-transfected K-HOS cells. Bars indicate the means ± SD of three independent experiments, each performed in duplicate (*p < 0.05). **(G)** Zymography analysis of conditioned media was performed after 48 h of serum-free cultures of YAPS127A-, YAPS94A-, and mock-transfected K-HOS cells. A Coomassie blue-stained gel that represents three independent experiments is shown (left panel).

To further explain the role of YAP in the metastatic process, we compared the morphology of YAPS127A-, YAPS94A-, and mock-transfected OS cells by analyzing F-actin cytoskeleton and focal adhesions, revealed by phalloidin labeling and vinculin immuno-staining, respectively. As shown in **Figure 2A** and **Supplementary Figure S1C**, cells expressing YAP-mutated proteins (YAPS127A- or YAPS94A-transfected cells) show a similar profile of elongated cells, distinct from mock-transfected cells, with increased actin stress fibers and focal adhesions at the cell front. We therefore observe an increase in the ratio of long axis length to short axis length of the cell body when YAP able to interact with TEAD or not is overexpressed (**Supplementary Figure S1D**). In addition, the number of focal adhesions was increased (**Supplementary Figure S1E**). This phenotype is indicative of the pro-migratory properties of YAP-transfected cells, regardless of the mutated form of YAP.

Against this background, GSEA analysis shows a statistically significant enrichment of focal adhesion-related genes in YAPS94A- and YAPS127A-transfected cells compared to mock-transfected cells. We can observe that the genes whose expression is modified are not the same according to the overexpression of the two mutated forms of YAP (YAPS94A- or YAPS127A-transfected cells). These results strongly suggests that two mechanisms, one TEAD dependent and the other TEAD independent, are involved in the YAP-driven expression of genes related to focal adhesion. The genes whose expression is increased by the expression of a YAP unable to interact with TEAD include genes coding for G-proteins such as *RHOB* and *RHOD* and chemokine receptors such as *CXCR4* or FAK stimulators such as *THBS1* (**Figure 2B** and **Supplementary Figure S1A**).

The process of epithelial-to-mesenchymal transition (EMT) of carcinoma exists as EMT-”like” in tumors of mesenchymal origin, such as OS, and confers to cancer cells an increase in their motility potential. Interestingly, the expression of genes involved in EMT-”like” in OS, such as *N-cadherin*, *Snail*, *Twist2*, and *vimentin*, is significantly increased in cells expressing YAP both able and unable to interact with TEAD (YAPS94A- or YAPS127A-transfected cells *versus* mock-transfected cells (**Figure 2C** and **Supplementary Figure S1B**). To determine whether the acquisition of this pro-migratory phenotype results in an increase of cell migration, we performed real-time migration assays. As shown in **Figure 2D**, both YAPS94A and YAPS127A overexpression significantly increased the ability of OS cells to migrate. To support these results, the GSEA analysis revealed that YAPS127A and YAPS94A overexpression is positively associated with genes involved in cell migration compared to mock-transfected cells. We can observe that the genes whose expression is modified are not the same according to the overexpression of the two mutated forms of YAP (YAPS94A- or YAPS127A-transfected cells) (**Figure 2E**). These results strongly suggest that two mechanisms, one TEAD dependent and the other TEAD independent, are involved in the YAP-driven expression of genes related to cell migration. Furthermore, the RT-qPCR analysis indicates that YAPS94A and YAPS127A overexpression induces the expression of *MMP-2* and *MMP-9*, two metalloproteinases that play a key role in the cell invasion process (**Figure 2F**). In addition, zymography assays demonstrate that YAPS94A and YAPS127A overexpression strongly stimulate the enzymatic activity of MMP-2 and MMP-9 (**Figure 2G**). Interestingly, we can note that the expression and activity of MMP-2 and MMP-9 are higher in YAP94A cells than in YAPS127A cells, suggesting that the modulation of the expression of these MMPs by the Hippo/YAP pathway does not require TEAD.

Taken together, these results demonstrate that two mechanisms, one TEAD dependent and the other TEAD independent, are involved in the YAP-driven EMT-like process and OS cell migration.

### 3.3 YAP Enhances TGF-β/Smad3 Transcriptional Activity in OS

Since (a) studies showed that Hippo and TGF-β/Smad3 signaling pathways are known to interact in the control of gene transcription (22) and (b) we previously proved that the TGF-β pathway plays a fundamental role in lung metastasis development in OS (23), we investigated the connection between these two pathways in the OS metastatic process.

**Figure 3:**
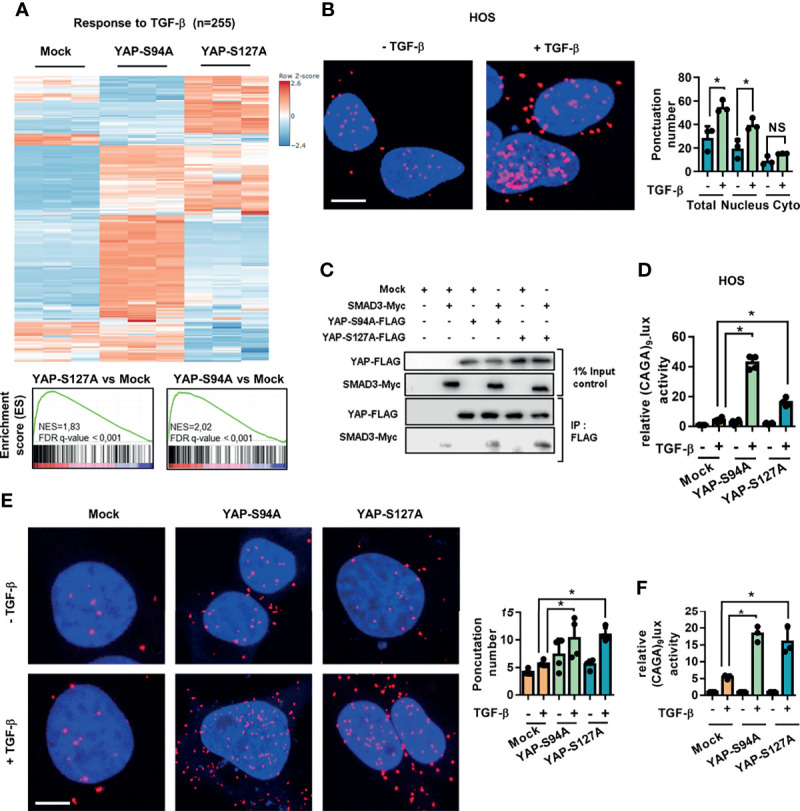
Regardless of its ability to bind TEAD, YAP drives TGF-β/Smad3 transcriptional activity. **(A)** Heat map showing the mRNA levels of TGF-β target genes. Enrichment score (ES) plots of gene set enrichment analysis indicate a significant upregulation of TGF-β target genes in YAPS94A- and YAPS127A-transfected K-HOS cells compared to mock-transfected cells. The color scales are based on Z-scores. **(B)** Localization of endogenous YAP/Smad3 complexes by *in situ* proximity ligation assay (PLA) in HOS cells in the presence or absence of TGF-β (5 ng/ml, 1 h). The red signal was obtained using Alexa555-labeled hybridization oligo nucleotides targeting amplified *in situ* PLA products. DAPI (blue) staining was used for nuclear visualization (left panel). Bars indicate the means ± SD of three independent experiments (*p < 0.05, right panel; NS, not significant). Bar = 10 µM. **(C)** HEK293 cells were co-transfected with YAPS94A-Flag, YAPS127A-Flag, Smad3-Myc, or empty vectors as indicated. At 24 h after transfection, the cells were treated with TGF-β (5 ng/ml during 1 h) and then subjected to immunoprecipitation with anti-Flag antibody followed by Western blotting by YAP and Smad3 antibodies as indicated. Representative photographs of two independent experiments are shown. **(D)** HOS cells were transiently transfected with the Smad3/4-specific construct (CAGA)_9_-lux with or without YAPS94A-, YAPS127A-, or empty vectors as indicated. At 24 h after transfection, TGF-β (5 ng/ml) was added for another 24 (h) Bars indicate the means ± SD of three independent experiments, each performed in duplicate (*p < 0.05). **(E)** Left panel: localization of YAP/Smad3 complexes by *in situ* PLA in mock-, YAPS94A-, and YAPS127A-stably transfected K-HOS cells in the presence or absence of TGF-β (5 ng/ml during 1 h). The red signal was obtained using Alexa555-labeled hybridization oligonucleotides targeting amplified *in situ* PLA products, and DAPI (blue) staining was used for nuclear visualization. Right panel: quantification of YAP/Smad3 complexes by *in situ* PLA in mock-, YAPS94A-, and YAPS127A-stably transfected K-HOS cells in the presence or absence of TGF-β (5 ng/ml during 1 h). Bars indicate the means ± SD of three independent experiments (*p < 0.05, right panel). Bar = 10 µM. **(F)** YAPS94A-, YAPS127A-, or mock- stably transfected K-HOS cells were transfected with the Smad3/4-specific construct (CAGA)_9_-lux. At 24 h after transfection, TGF-β (5 ng/ml) was added for another 24 (h) Bars indicate the means ± SD of three independent experiments, each performed in duplicate (*p < 0.05).

First, the GSEA analysis reveals a statistically significant enrichment of TGF-β pathway-regulated genes in the YAPS94A- and YAPS127A-transfected compared to mock-transfected cells (**Figure 3A**). In this context, we examined the relationship between YAP and Smad3, the main transcriptional effectors of TGF-β signaling, in a panel of three human OS cell lines: HOS, MG63, and G292 cells. The *in situ* PLA assays clearly establish an increase of nuclear YAP–Smad3 interactions in OS cell lines upon TGF-β stimulation (**Figure 3B** and **Supplementary Figure S2A**). We then studied the ability of YAP-mutated protein YAPS94A not able to interact with TEAD to interact with Smad3 protein. Immunoprecipitation assays demonstrate the interactions between YAPS94A and Smad3, revealing that the ability of YAP to interact with TEAD is not essential for the protein–protein interaction between YAP and Smad3 (**Figure 3C**). Furthermore, YAPS94A and YAPS127A both increase the ability of TGF-β to trans-activate the Smad3/4-specific reporter construct (CAGA)_9_-lux in OS cells (**Figure 3D** and **Supplementary Figure S2B**). Furthermore, the use of siRNA directed against TEAD confirms that this transcription factor is not essential for YAP-driven TGF-β transcriptional response (**Supplementary Figure S2C**). To elucidate a potential role of TGF-β/Smad3 signaling in YAP-driven OS metastatic process, we then probed the consequences of the stable overexpression of the YAPS127A- and YAPS94A-mutated proteins, both of which are able to interact with Smad3, in K-HOS cells. The *in situ* PLA assays demonstrate an increase of TGF-β-driven YAP–Smad3 interactions in the nucleus of YAPS94A- and YAPS127A-transfected cells compared with the number of interactions measured in mock-transfected cells (**Figure 3E**). In addition, the YAPS94A-transfected cells exhibit a strong increase of TGF-β-transcriptional response as measured by transient cell transfection experiments with the Smad3/4-specific reporter construct (CAGA)_9_-lux (**Figure 3F**).

These results together demonstrate that YAP drives TGF-β/Smad3 transcriptional activity regardless of its ability to bind TEAD.

### 3.4 OS Cell Migration and *In Vivo* Metastatic Process Depend on the Functional Interaction Between the Hippo/YAP and TGF-β/Smad3 Signaling Pathways

**Figure 4:**
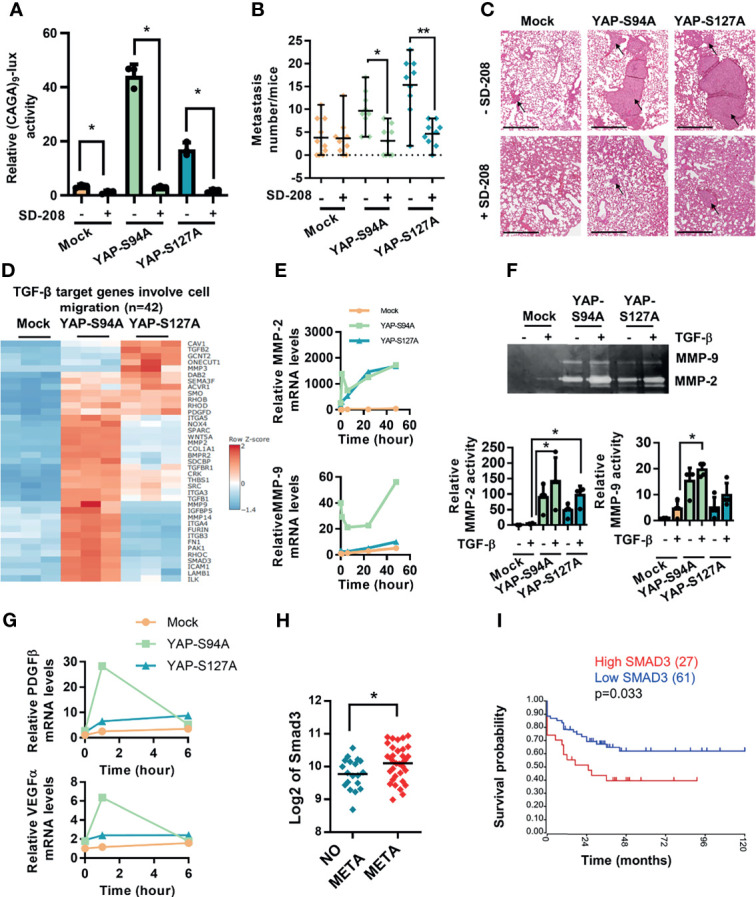
Crucial role of TGF-β signaling in YAP-induced osteosarcoma (OS) cell migration and *in vivo* OS metastatic process. Correlation between Smad3 gene expression and overall survival of OS patients. **(A)** Cells were transfected with the Smad3/4-specific construct (CAGA)9-lux. At 24 h after transfection, TGF-β (5 ng/ml) was added during 24 h in the presence or absence of SD-208 (10 ng/ml) for another 24 (h) Bars indicate the means ± SD of three independent experiments, each performed in triplicate (*p < 0.05). **(B)** Intramuscular paratibial injections of 1.106 mock-, YAPS94A-, and YAPS127A-transfected K-HOS cells were performed in six groups of 10 nude mice and treated with vehicle or SD-208 (60 mg/kg/day) as indicated. The mice were sacrificed when the tumor sizes reached 1,000 mm^3^, and the lungs were removed. Number of lung metastases (mean ± SD; *p < 0.05, **p < 0.01). **(C)** Representative hematoxylin and eosin staining of representative lungs for each group. Bar = 500 µm. **(D)** Heat map of TGF-β target genes involved in the cell migration process in mock-, YAPS94A-, and YAPS127A-transfected K-HOS cells as analyzed by RNA-seq. The color scales are based on Z-scores. **(E)** MMP-2 and MMP-9 mRNA steady-state levels were quantified by RT-q-PCR analysis in mock-, YAPS94A-, and YAPS127A-transfected K-HOS cells in the presence or absence of TGF-β (5 ng/ml during 6, 12, 24, and 48 h). Bars indicate the means ± SD of three independent experiments, each performed in duplicate. **(F)** Zymography analysis of conditioned media was performed after 48 h of serum-free cultures of YAPS127A-, YAPS94A-, and mock-transfected K-HOS cells in the presence or absence of TGF-β (5 ng/ml). A representative of Coomassie blue-stained gel of three independent experiments is shown (upper panel). Quantification of relative MMP-2 and MMP-9 activities (lower panels). Bars indicate the means ± SD of three independent experiments (*p < 0.05). **(G)** PDGFβ and VEGFα mRNA steady-state levels were quantified by RT-q-PCR analysis in mock-, YAPS94A-, and YAPS127A-transfected K-HOS cells in the presence or absence of TGF-β (5 ng/ml for 6, 12, 24, and 48 h). Bars indicate the means ± SD of three independent experiments, each performed in duplicate. **(H)** Relative Smad3 gene expression in OS patients with or without metastases at diagnosis following bioinformatics analysis of RNAseq data seq GSE42352 (21) from an OS patient cohort comprising 53 samples. Analysis was done using R2 (http://r2.amc.nl). *p < 0.05. **(I)** Kaplan–Meier analysis of the survival outcomes of 88 patients dichotomized into high level and low level of Smad3 expression, following the analysis of the RNAseq dataset GSE42352 (21). The analysis was performed using R2 (http://r2.amc.nl).

To validate the biological relevance of our findings, we next investigated whether the inhibition of the TGF-β/Smad3 cascade in OS cells could alter the capacity of YAP to enhance lung metastasis development. Therefore, we used SD208, an ALK5 inhibitor previously described as an inhibitor of the TGF-β/Smad3 signaling pathway in OS cells (24), and showed, as anticipated, that SD-208 efficiently blocks the ability of TGF-β to transactivate the Smad3/4-specific reporter construct (CAGA)_9_-lux and the ability of YAPS94A and YAPS127A to increase TGF-β responsiveness (**Figure 4A**). Interestingly, although SD-208 does not affect the growth of the primary tumor, it blocks the ability of both YAPS94A- and YAPS127A-mutated proteins to increase the number of lung metastases in mice models (**Figures 4B, C** **)**, demonstrating the crucial role of TGF-β/Smad3 signaling pathway in YAP-driven lung metastasis development. In this context, the RNAseq analysis reveals a group of genes overexpressed in YAPS94A- and YAPS127A-transfected cells that are both identified as TGF-β targets and implicated in cell migration (**Figure 4D**).

Furthermore, RT-qPCR and zymography analyses indicate that YAPS94A and YAPS127A overexpression increases the ability of TGF-β to stimulate the expression (**Figure 4E**) and activation (**Figure 4F**) of MMP-2 and MMP-9, two metalloproteases implicated in the invasion process. In addition, the RT-qPCR analysis indicates that YAPS94A and YAPS127A overexpression enhances the ability of TGF-β to stimulate the expression of VEGF and PDGF, two cytokines that play a crucial role in the angiogenesis process (**Figure 4G**). It can be noted that the response to TGF-β is strongly enhanced, in particular, by the overexpression of a YAP protein unable to interact with TEAD.

Finally, to determine the clinical significance of the role played by Smad3 in OS tumor progression, we investigated Smad3 gene expression using data extracted from the GSE21257 database. While we previously showed increased nuclear localization of Smad3 in biopsies from OS patients with metastases at diagnosis (24), high Smad3 transcript is also significantly correlated with the presence of metastases (**Figure 4H**). In addition, high Smad3 gene expression predicts a poor survival outcome in OS patients, as illustrated in the Kaplan–Meier plot in **Figure 4I**.

Taken together, these outcomes demonstrate (a) that the TGF-β/Smad3 signaling pathway plays a crucial role in a YAP able or not to interact with TEAD to drive OS cell migration and *in vivo* lung metastasis development and (b) a correlation between the expression of Smad3 and both the presence of metastases and the survival outcomes of OS patients.

### 3.5 Verteporfin Inhibits OS Lung Metastatic Development

To confirm YAP as a potential therapeutic target for OS treatment, we finally assessed the effect of verteporfin, a Hippo/YAP inhibitor, on lung metastasis development in the preclinical model of OS.

**Figure 5:**
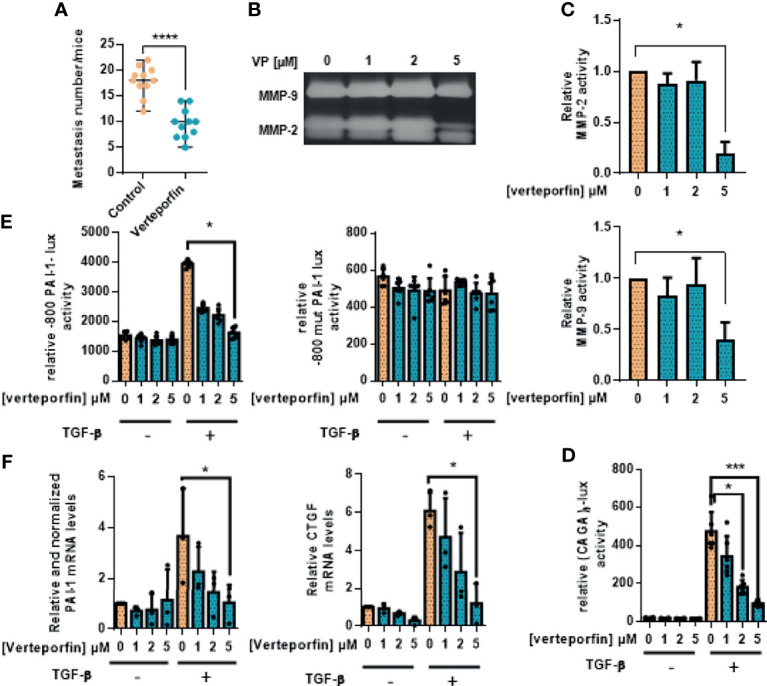
Verteporfin prevents osteosarcoma primary bone tumor and lung metastatic spreading. **(A)** Intramuscular paratibial injections of 1.10^6^ HOS cells were done in two groups of 12 nude mice treated with vehicle or verteporfin (20 mg/kg/day) as indicated. The mice were sacrificed when the tumor sizes reached 2,000 mm^3^, and the lungs were removed. The number of lung metastases (mean ± SD; ****p < 0.001). **(B)** Zymography analysis of conditioned media from 48-h serum-free cultures of HOS cells treated with verteporfin as indicated. A Coomassie blue-stained gel representative of three independent experiments is shown. **(C)** Relative MMP-2 and MMP-9 activity. Bars indicate mean ± SD of three independent experiments; *p < 0.05. **(D)** HOS cells were transfected with the Smad3/4-specific construct (CAGA)_9_-lux. At 24 h after transfection, the cells were treated for 24 h with or without verteporfin as indicated and were treated or not with TGF-β (5 ng/ml, 24 h). Bars indicate the mean ± SD of at least three independent experiments carried out in triplicate (*p < 0.05, ***p < 0.005). **(E)** HOS cells were transfected with the -800PAI-1-lux construct mutated (-800mutPAI-1-lux) or not (-800PAI-1-lux) on CAGA sequences (-800mutPAI-1-lux). At 24 h after transfection, the cells were treated for 24 h with verteporfin as indicated and with or without TGF-β (5 ng/ml, 24 h). Bars indicate the mean ± SD of at least three independent experiments carried out in triplicate (*p < 0.05). **(F)** HOS cells were pretreated for 24 h with verteporfin as indicated and were treated or not with TGF-β (5 ng/ml, 6 or 48 h). After incubation, the mRNA steady-state levels of the specific TGF-β target genes *CTGF* (6 *h*) and PAI-1 (48 h) were measured by quantitative RT-PCR. Bars indicate the means ± SD of at least three independent experiments, each performed in triplicate (*p < 0.05).

We have previously shown that verteporfin blocks the YAP/TEAD signaling pathway, as it inhibits the transactivation of the TEAD-specific reporter construct (TEAD)_8_-lux and the expression of *CYR61*, a target gene of YAP/TEAD [(14), see **Figure 5**]. Experiments using the preclinical experimental model of OS show that the number of lung metastases is dramatically reduced in the verteporfin-treated mice group (**Figure 5A**). To better understand this result, zymography assays demonstrate that verteporfin inhibits the activation of HOS cell-secreted MMP-2 (**Figure 5B**). Since we demonstrated that the TGF-β/Smad3 signaling pathway plays a crucial role in the YAP-driven lung metastatic process in OS, we next examined the effect of verteporfin on the TGF-β/Smad3 cascade. Interestingly, verteporfin decreases the ability of TGF-β to transactivate the Smad3/4-specific reporter constructs (CAGA)_9_-luc and -800PAI-luc (**Figures 5C, D** **)** and to increase the expression of two TGF-β/Smad3 target genes, *PAI-1* and *CTGF* (**Figure 5E**).

Taken together, these results propose that the Hippo/YAP signaling pathway could represent a therapeutic target against OS lung metastases.

## 4 Discussion

Tumorigenesis is a multi-stage process that most often leads to the development of metastases. Concerning OS, primary tumor grows foremost in the metaphysis of the long bone and is inclined to distant metastases, with the lungs being the most frequent site (25). Despite the improvement in prognosis due to the application of chemotherapy protocols that mainly control primary tumor growth, the 5-year survival rates of patients with lung metastasis remain dramatically low (26, 27). Therefore, further exploration of the mechanisms underlying lung metastasis seems to be of importance.

High YAP expression and/or YAP activation have been described in several solid tumor types and linked with poor prognosis (13). It has been suggested that YAP acts as an oncogene through the activation of target genes able to stimulate proliferation, EMT, and metastases development (11, 13, 28, 29). Although YAP is of emerging importance in many cancers, the exact mechanisms underlying its crucial functions in cancer progression have not yet been elucidated. While YAP/TEAD axis was reported in the control of OS primary tumor growth (14), the molecular mechanisms underlying YAP-driven OS lung metastatic development have not been established.

In this work, overexpression of a YAP mutant protein able (YAPS127A) or unable (YAPS94A) to interact with TEAD proteins in OS cells is found to stimulate the development of *in vivo* lung metastases. A key aspect of the metastasis process is the EMT multi-step program promoting the migratory and invasive properties of cancer cells (30). During the last few years, a crosstalk between the YAP cascade and EMT transcriptional factors such as ZEB1/2, Snail/Slug, and Twist has been described in several carcinomas (13). Here we clearly demonstrated that the overexpression of YAP proteins, regardless of their ability to bind TEAD, stimulates the expression of the mesenchymal markers N-cadherin and vimentin and the expression of the EMT transcriptional factors Snail and twist2, suggesting that TEADs are not fully necessary for the YAP-driven EMT-”like” process in OS. We can thus speculate that crosstalks between the YAP cascade and other signaling pathways widely implicated in EMT, such as TGF-β/Smad cascades, drive the EMT-”like” process in OS (23). Finally, functional assays revealed that YAP overexpression stimulates OS cell migration and MMP-2/9 activity, two MMPs implicated in the tumor metastatic process such as in OS (25).

Remodeling of actin cytoskeleton is clearly associated with the ability of cells to migrate and invade. In this context, many published works reported that YAP nuclear activity is correlated with the stability of actin cytoskeleton, which is mainly controlled by the RhoGTPase pathways (31). Here we specifically demonstrate that the overexpression of YAP protein induces an OS cell pro-migratory phenotype associated with the expression of genes linked to both cell migration and focal adhesion assemblies such as THBS1. In accordance with previous works demonstrating that YAP regulates cell migration by controlling focal adhesion assembly (32, 33), we can speculate that an important subset of the YAP transcriptional program is devoted to installing a positive feedback loop that promotes actin remodeling and therefore stimulates migratory activity in OS.

We thus clearly demonstrate that overexpression of YAP protein, able or not to interact with TEAD, drives the ability of OS cells to migrate and thus stimulate the lung metastatic process.

It is now widely accepted that TGF-β acts both as a tumor suppressor in premalignant carcinoma and as a tumor promoter in advanced carcinoma (23). Regarding OS, we recently demonstrated that TGF-β only exerts pro-tumoral activities, specifically by stimulating the ability of OS cells to migrate and invade and thus by stimulating the development of pulmonary metastases (23, 24). In this context, we formulated the hypothesis that YAP drives the OS metastatic process in part *via* the TGF-β/Smad3 signaling pathway. We clearly demonstrate that blocking the TGF-β/Smad3 cascade by using the TβRI inhibitor SD-208 inhibits the ability of YAP protein to drive lung metastasis development in OS, regardless of its binding to TEAD. One of the first studies that described the involvement of YAP/TAZ and TGF-β signaling in nucleocytoplasmic shuttling indicated that TAZ interacts with Smad2/4 and Smad3/4 complexes in epithelial cells (34). Similar findings were observed for YAP, which forms a complex with Smad3 in mesothelial cells (35) and in skin epithelial cells (36). Here we clearly demonstrate that a YAP mutant protein not able to interact with TEAD (YAPS94A) interacts with Smad3 and therefore stimulates Smad3/4 transcriptional activity in OS. Notably, high levels of TEAD binding-incompetent YAP drastically enhance the capacity of TGF-β to stimulate key functions implicated in the metastatic process, such as cell migration. We further performed transcriptomic analysis in OS cells to allow the identification of TEAD-independent modulation of YAP target genes associated with TGF-β responsiveness. Finally, an interesting observation that strongly supports the crucial role of TGF-β/Smad3 in the YAP-driving metastatic process in OS is that Smad3 is highly expressed in OS patients with metastases at diagnosis and is associated with poor survival outcomes. Although our work demonstrates the interaction between Smad3 and YAP and thus the role of Smad3 in the ability of YAP to stimulate the metastatic progression of OS by stimulating EMT and OS cell migration, we cannot exclude the role of another mediator of the TGF-β pathway, Smad2, which is able to interact with TAZ, an analogue of YAP (34).

To confirm YAP as a potential therapeutic target in metastatic OS, we finally tested the effect of verteporfin on OS lung metastasis development. Verteporfin is a light-activated drug used in photodynamic therapy for the treatment of choroidal neovascular membranes (37). We report here that verteporfin inhibits lung metastasis development and efficiently blocks the TGF-β/Smad3 transcriptional activity. Originally reported as an inhibitor of the YAP/TEAD interaction (37), verteporfin was recently reported as able to induce the degradation of YAP protein in OS cell lines (14), demonstrating its aptitude to target the YAP signaling pathway *via* different mechanisms (38). We can thus speculate that verteporfin inhibits OS lung metastasis development *via* its ability to both inhibit YAP-driven TGF-β/Smad3 transcriptional and YAP-driven TEAD transcriptional activities.

**Figure 6:**
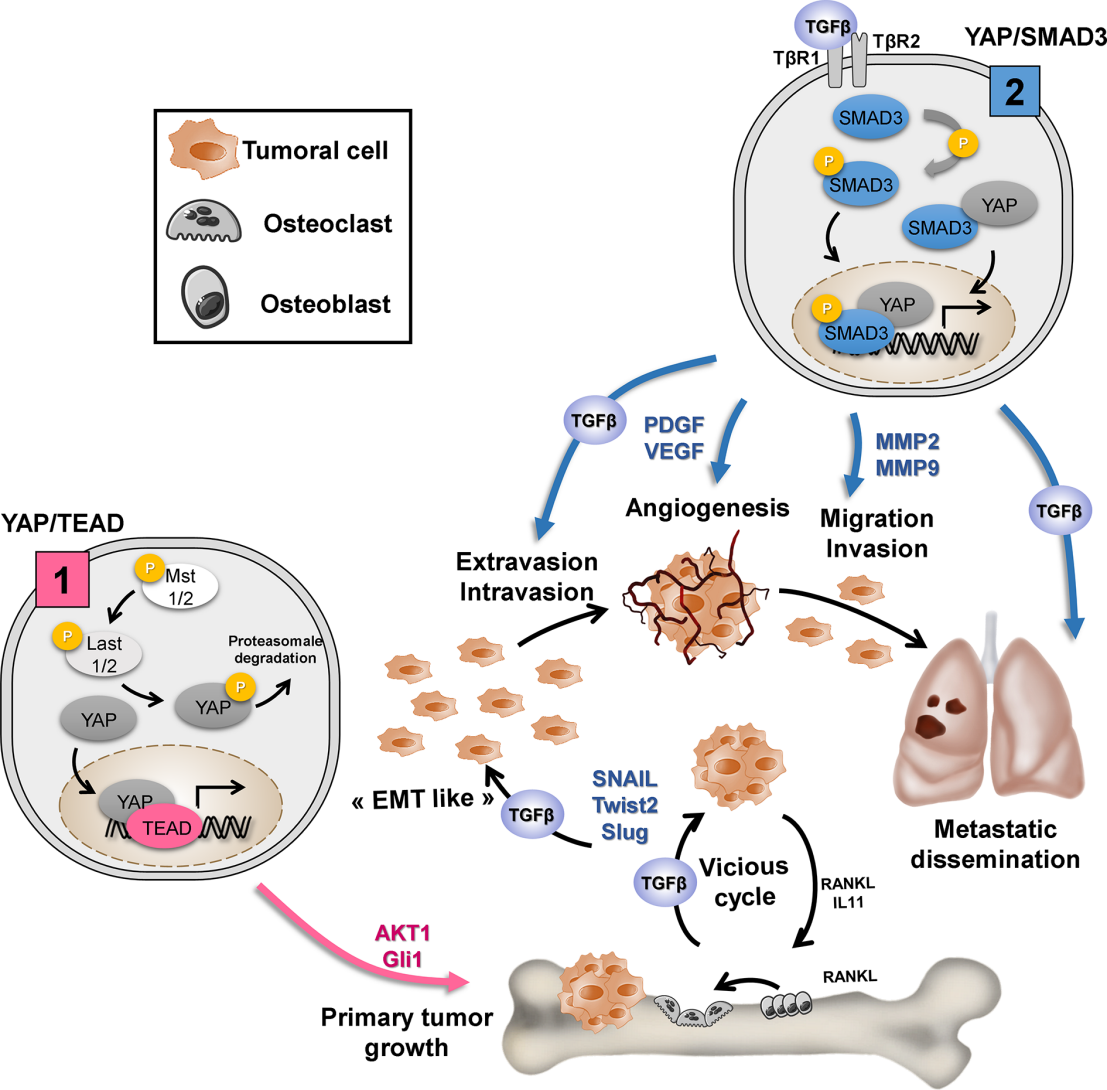
Depending on the transcription factor to which it will bind, YAP regulates the key functions of tumor development. (1) TEAD is crucial for YAP-driven cell proliferation and primary tumor growth: YAP/TEAD stimulates cell proliferation by regulating the expression of genes directly involved in cell cycle control or by regulating the expression of mediators of other signaling pathways involved in the cell cycle, such as Gli or AKT. (2) TGF-β/Smad3 is crucial for YAP-driven lung metastasis development: TGF-β stimulates “EMT-like”, cell migration and invasion in part by increasing MMP-2/MMP-9 expression. TGF-β upregulates the expression of osteolytic factors such as RANKL (receptor activator of nuclear factor kappa-B ligand) and IL-11 (interleukin-11) and therefore stimulate bone osteolysis and the secretion of protumoral factors. TGF-β upregulates PDGF (platelet-derived growth factor) and VEGF (vascular endothelial growth factor) expression, and therefore angiogenesis.

The integration of several signaling pathways by a central actor within tumor cells represents an attractive strategy for treating both tumor growth and the metastatic process. Recently, we show that the YAP/TEAD axis is a central actor in OS primary tumor growth (14), and we here demonstrated that the YAP/TGF-β axis is involved in YAP-driven OS lung metastasis development. It thus appears that YAP regulates key functions of tumor development *via* binding to different transcriptional factors. We propose that (1) TEAD is crucial for YAP-driven key functions associated with primary tumor growth such as cell proliferation and (2) Smad3 alone or in partnership with TEAD is crucial for YAP-driven key functions associated with the metastatic process, such as EMT, migration, and invasion (**Figure 6**).

## Data Availability Statement

The datasets supporting the present study are deposited in the public repository GEO, accession number GSE184003 (https://www.ncbi.nlm.nih.gov/geo/query/acc.cgi?acc=GSE184003).

## Ethics Statement

The animal study was reviewed and approved by the French Ethical Committee (CEEA Pays de la Loire n°06: project authorization 8405).

## Author Contributions

SM and FV contributed to the conceptualization. SM, GD, MM, and RT contributed to the methodology. SM and RT contributed to the formal analysis. SM, GD, MM, RB, IC, and MD participated in the investigation. SM, IC, and FV contributed to writing—original draft. BO, BB-LR, and FR contributed to writing—review and editing. FV supervised the study. SM and FV participated in funding acquisition. All authors contributed to the article and approved the submitted version.

## Funding

This work was supported by Imagine for Margo, Societé Française de lutte contre les cancers et les leucémies de l’enfant et de l’adolescent, l’étoile de Martin, Enfants Cancers Santé, Ligue contre le cancer (comité 44, 49 et 85). This work was also supported by “Cancéropole Grand Ouest, Réseau Molécules marines, métabolisme et cancer”.

## Conflict of Interest

The authors declare that the research was conducted in the absence of any commercial or financial relationships that could be construed as a potential conflict of interest.

## Publisher’s Note

All claims expressed in this article are solely those of the authors and do not necessarily represent those of their affiliated organizations, or those of the publisher, the editors and the reviewers. Any product that may be evaluated in this article, or claim that may be made by its manufacturer, is not guaranteed or endorsed by the publisher.
