# Supplementary material for: Involvement of the TGF-β Signaling Pathway in the Development of YAP-Driven Osteosarcoma Lung Metastasis
Source: Front Oncol. 2021 Oct 26;11:765711. doi: 10.3389/fonc.2021.765711 (PMC8576330; doi:10.3389/fonc.2021.765711)
Supplement: Supplementary file 1 [file DataSheet_1.docx]

Supplementary Material

## Supplementary Figures


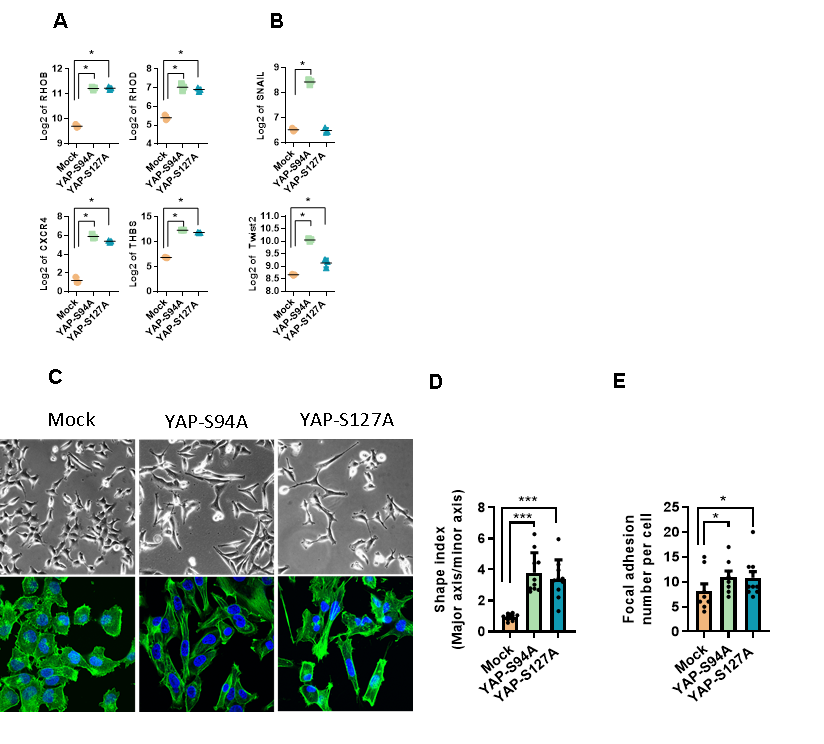


**Supplementary Figure 1: Role of YAP in the control of cell migration**

(**A**) Median Log2(FPKM) expression of RHOC, RHOD, THBS1 and CXCR4 between mock-, YAPS94A- and YAPS127A- transfected cells following bioinformatics analysis of RNA sequencing (**B**) Median Log2(FPKM) expression of Twist2 and Snail genes expression in mock-, YAPS94A- and YAPS127A- transfected cells following bioinformatics analysis of RNA sequencing. (**C**) Upper panel: photograph of cultures of mock-, YAPS94A- and YAPS127A- transfected cells. Lower panel: of cultures of mock-, YAPS94A- and YAPS127A- transfected cells were fixed, permeabilized, F-actin cytoskeleton and nuclei were respectively revealed by phalloidine (red) and DAPI labelling (blue). (**D**) The ratio of long axis length to short axis length of the cell body was quantified. Bars indicate means ± S.D. for ten cells (***p< 0.001). (**E**) The number of focal adhesions per cell was counted. Bars indicate means ± SEM. (*p< 0.05).

**
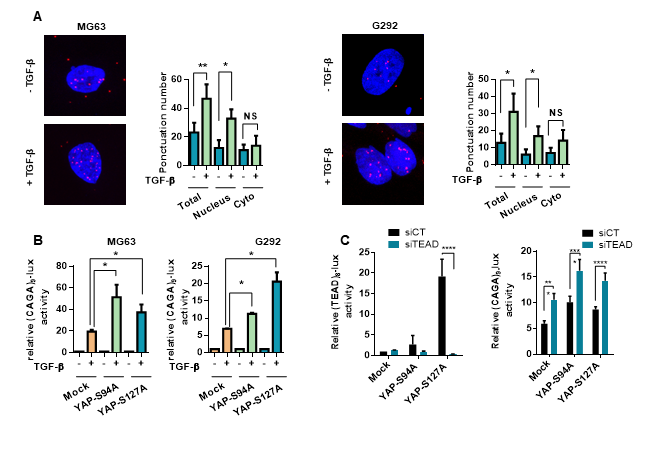
**

**Supplementary Figure 2 YAP drives TGF-β/Smad3 transcriptional activity regardless of its ability to bind TEAD.**

(**A**) Localization of endogenous YAP/Smad3 complexes by in situ PLA in MG63 and G292 cells in presence or absence of TGF-β (5 ng/ml during 1h). The red signal was obtained using Alexa555-labeled hybridization oligo nucleotides targeting amplified in situ PLA products. DAPI (blue) staining was used for nuclear visualization (left panel). Bars indicate means ± S.D. of three independent experiments (*P < 0.05, **P < 0.01).(**B**) MG63 and G292 cells were co-transfected with the Smad3/4-specific construct (CAGA)_9_-lux and either the YAPS94A, YAPS127A, or empty vectors. 24h after transfection TGF-β (5 ng/ml) was added during 24h. Bars indicate means ± S.D. of three independent experiments, each performed in triplicate (*p< 0.05).(**C**) HOS cells were co-transfected with or without 1) the siRNA against TEAD (siTEAD) or control siRNA (siCT) and with, 2) the YAPS94A, YAPS127A, or empty vectors, and 3) the TEAD-specific reporter construct (TEAD)8-lux (left panel) and Smad3/4-specific construct (CAGA)9-lux (right panel) 24h after transfection TGF-β (5 ng/ml) was added or not during 24h. Bars indicate means ± S.D. of three independent experiments, each performed in triplicate (**p< 0.01, ***p< 0.001, ****p< 0.0001)
